# Identification of an Endogenous Ligand Bound to a Native Orphan Nuclear Receptor

**DOI:** 10.1371/journal.pone.0005609

**Published:** 2009-05-19

**Authors:** Xiaohui Yuan, Tuong Chi Ta, Min Lin, Jane R. Evans, Yinchen Dong, Eugene Bolotin, Mark A. Sherman, Barry M. Forman, Frances M. Sladek

**Affiliations:** 1 Department of Gene Regulation and Drug Discovery, Gonda Diabetes Research Center, The Beckman Research Institute at the City of Hope National Medical Center, Duarte, California, United States of America; 2 Cell, Molecular and Developmental Biology Graduate Program, University of California Riverside, Riverside, California, United States of America; 3 Department of Cell Biology and Neuroscience, University of California Riverside, Riverside, California, United States of America; 4 Genetics, Genomics and Bioinformatics Graduate Program, University of California Riverside, Riverside, California, United States of America; 5 Department of Biomedical Informatics, The Beckman Research Institute at the City of Hope National Medical Center, Duarte, California, United States of America; Ecole Normale Supérieure de Lyon, France

## Abstract

Orphan nuclear receptors have been instrumental in identifying novel signaling pathways and therapeutic targets. However, identification of ligands for these receptors has often been based on random compound screens or other biased approaches. As a result, it remains unclear in many cases if the reported ligands are the true endogenous ligands, – i.e., the ligand that is bound to the receptor in an unperturbed *in vivo* setting. Technical limitations have limited our ability to identify ligands based on this rigorous definition. The orphan receptor hepatocyte nuclear factor 4 α (HNF4α) is a key regulator of many metabolic pathways and linked to several diseases including diabetes, atherosclerosis, hemophilia and cancer. Here we utilize an affinity isolation/mass-spectrometry (AIMS) approach to demonstrate that HNF4α is selectively occupied by linoleic acid (LA, C18:2ω6) in mammalian cells and in the liver of fed mice. Receptor occupancy is dramatically reduced in the fasted state and in a receptor carrying a mutation derived from patients with Maturity Onset Diabetes of the Young 1 (MODY1). Interestingly, however, ligand occupancy does not appear to have a significant effect on HNF4α transcriptional activity, as evidenced by genome-wide expression profiling in cells derived from human colon. We also use AIMS to show that LA binding is reversible in intact cells, indicating that HNF4α could be a viable drug target. This study establishes a general method to identify true endogenous ligands for nuclear receptors (and other lipid binding proteins), independent of transcriptional function, and to track *in vivo* receptor occupancy under physiologically relevant conditions.

## Introduction

Nuclear receptors (NRs) are ligand-dependent transcription factors that regulate the expression of genes involved in virtually all aspects of physiology and disease [1], [2]. The identification of ligand-receptor pairs began with the pioneering work of Jensen [3], Edelman [4] and others who injected radioactive ligands into animals and observed their binding to nuclear receptor proteins. These experiments had an inherent bias in that the probe ligand displaced binding of the true endogenous ligand, which could not be detected in these assays. The validity of the classical steroid receptor-ligand pairs are now well established, but the limitations of existing approaches leave open the possibility that additional ligands may exist for the classical steroid receptors. Indeed, such speculation was raised for estrogen receptor some time ago, as well as more recently [5], [6], and for intestinal vitamin D receptor that is activated by an enterohepatic bile acid [7].

The assignment of endogenous ligands to the so-called orphan nuclear receptors is even more equivocal [2], [8]. Typically, orphan receptors are screened in transcription-based assays against random compound collections that include natural or synthetic molecules; in other cases candidate ligands are identified based on their structure or biological activity [9]. Alternatively, compounds are added at supra-pharmacologic doses and their metabolic products are found to be ligands [10]. Most recently, ligands have been reported based on fortuitous binding to heterologously produced recombinant proteins [11]–[17]. Such studies have been of enormous value and have lead to the identification of many novel signaling pathways, drugs and therapeutic targets. Nonetheless, ligands have been identified for only about half of the 48 human nuclear receptors and the tally in non-human species is even lower. Furthermore, it remains unclear how many of the ligands that have been identified are the actual ligands that are bound to the receptor *in vivo*. For example, the first orphan receptor-ligand pair to be identified was 9-cis retinoic acid and RXR [10], yet it appears unlikely that sufficient 9-cis retinoic acid exists *in vivo* to serve as a true endogenous ligand [18].

HNF4α (*HNF4A*, NR2A1) is another orphan receptor whose endogenous ligand remains unclear [19]–[21]. HNF4α is essential to early development and plays critical roles in hepatocyte differentiation [22]–[24] and in homeostasis of the adult liver, intestine, and pancreatic beta cells [25]–[28]. In humans, mutations in the coding and promoter regions of HNF4α lead to Maturity Onset Diabetes of the Young 1 (MODY1), a heritable form of type 2 diabetes [29]. Recent crystallographic studies identified a mixture of tightly bound fatty acids in the ligand binding pocket (LBP) of bacterially expressed HNF4α and HNF4γ [30], [31], but it remains unclear what ligands are bound when the receptor is expressed in its native physiological environment. There have also been somewhat controversial reports of fatty acyl Co-enzyme A thioesters as HNF4α ligands [32], [33].

These studies highlight the need to distinguish between ligands that may bind under non-native conditions and those that are the true endogenous ligands. The most rigorous definition of a true endogenous ligand is a compound that binds the LBP *in vivo* in the absence of experimental probes or other perturbations. Identification of ligands by this definition requires new technical approaches.

In addition to identification of endogenous ligands for orphan (and other) nuclear receptors, new experimental tools to identify ligands are also needed to address the role of ligands in the evolution of the nuclear receptor superfamily [34]–[36]. Examination of HNF4 is also instructive in this regard as it is present in the earliest metazoan organisms and is one of the most evolutionarily conserved nuclear receptors [37], [38]. Therefore, the question of whether HNF4 binds an endogenous ligand, the identity of that ligand and its effect on HNF4 transcriptional activity is of particular interest and may be relevant to the entire receptor superfamily. However, these issues cannot be addressed without an assay that can identify potential ligands in the absence of a pre-supposed function.

Here we use an affinity isolation/mass spectrometry (AIMS) approach to identify the endogenous ligand that is bound to HNF4α in mammalian cells and in mouse liver. The approach is unbiased in that it does not make any pre-assumptions as to what the ligand might be or how it might affect HNF4α function. Our results indicate that the vast majority of HNF4α is bound to a single essential fatty acid: linoleic acid (LA). Furthermore, our results show that the binding is reversible, indicating that LA is an exchangeable ligand. To our knowledge, this represents the first time an endogenous ligand has been identified by virtue of its association with a nuclear receptor in animal tissue.

## Results

### Identification of an endogenous mammalian HNF4α ligand

**Figure 1:**
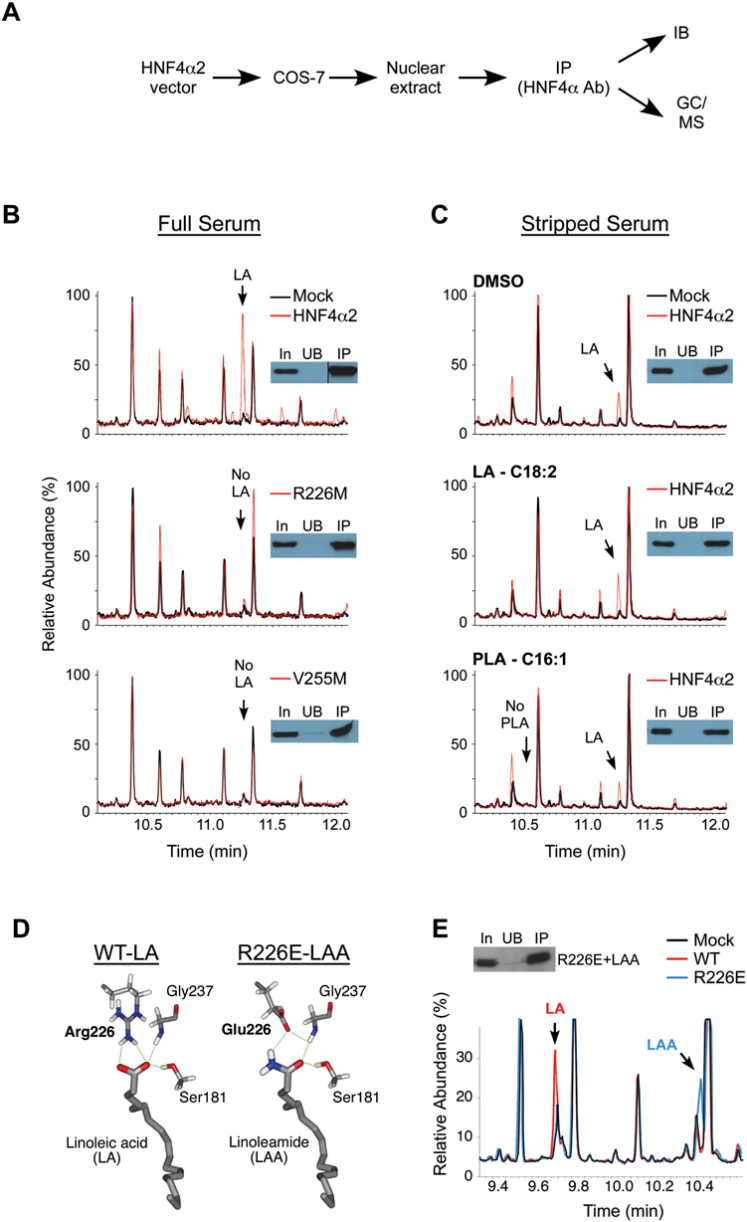
Identification of linoleic acid (LA) as the endogenous ligand for mammalian-expressed HNF4α2. (A) Affinity isolation/mass-spectrometry (AIMS): rat HNF4α2 was purified by immunoprecipitation (IP) from crude nuclear extracts of transfected COS-7 cells using an HNF4α specific antibody (HNF4α Ab). The amount of HNF4α2 protein recovered was determined by immunoblot (IB) analysis and bound ligands bound were identified by GC/MS. (B) GC/MS chromatograms (10 to 12 min) comparing compounds extracted from IP'd material from mock-transfected (black) to HNF4α2-transfected (red) (wt and LBP mutants R226M and V255M) COS-7 cells grown in 10% bovine calf serum (Full Serum). Insets: HNF4α IB showing input (In, 2% of total), material not bound by the Protein A Sepharose (unbound, UB, 2%), and IP'd material (IP, 4%). (C) Same as in (B) except lipid-depleted serum (Stripped Serum) was used and vehicle (DMSO), linoleic acid (LA, 30 µM) or palmitoleic acid (PLA, 30 µM) were added to the media as indicated. Arrow, position of LA and PLA peak as determined by standards. (D) Predicted model of HNF4α2 (WT) with bound LA showing the hydrogen bonding between the Arg226 guanidinium group and the LA carboxylate head group (left panel). Right panel, model of HNF4α R226E mutant bound to linoleamide (LAA) with the carboxylate group of the Glu226 mutant interacting with the LAA NH_2_ head group. (E) GC/MS chromatogram comparison and corresponding IB of HNF4α2 WT (red) and R226E mutant (blue) IP'd from COS-7 cells treated with 30 µM LAA. LA and LAA peaks are indicated.

To identify an endogenous ligand for HNF4α, we utilized an affinity isolation/mass-spectrometry (AIMS) approach outlined in Figure 1A. Wild type (wt) rat HNF4α2 was expressed in mammalian COS-7 cells and gas chromatography/mass spectrometry (GC/MS) was used to analyze the compounds bound to HNF4α2 immunoprecipitated (IP'd) from nuclear extracts. We found that HNF4α2 was associated with linoleic acid (LA, 9,12-octadecadienoic acid, C18:2-Δ9,12) (Figure 1B, top panel), an essential dietary polyunsaturated fatty acid not typically found in *E. coli*. Quantification of the levels of LA and wt HNF4α2 protein indicated that 30–100% of the receptor is occupied by LA, depending on the experiment. Although the presence of trace amounts of other fatty acids cannot be excluded, the only peak clearly identified was that of LA. These findings suggest that LA represents the predominant fatty acid associated with HNF4α in mammalian cells. Although unlikely, a trivial explanation for these findings is that LA is not bound to HNF4α within cells, but becomes associated with the receptor after lysis. To rule out this possibility, deuterated LA ([^2^H]LA) was added to the lysed cells and AIMS was performed as in Figure 1A. As expected, HNF4α was found associated with cellular [^1^H]LA but not with buffer-specific [^2^H]LA (see Supplementary Figure S1), indicating that the HNF4α•LA complex forms in cells prior to lysis.

To determine the specificity of LA binding, we asked whether LA, or other fatty acids, were bound to HNF4α proteins containing mutations in the LBP. We first mutated a key arginine residue (R226) that establishes a critical charge-charge interaction with the carboxylic acid moiety of the fatty acid [30], [31]. This R226M mutant did not bind LA (Figure 1B, middle), indicating that binding is specific to the LBP. Similarly, a V255M mutation that is found in patients with MODY1 [39], [40] also failed to bind LA (Figure 1B, bottom). Taken together, these findings demonstrate that LA specifically binds to the HNF4α LBP within mammalian cells.

We next examined whether LA could be removed or replaced from the HNF4α LBP by manipulating the media conditions. In cells grown in lipid-depleted serum, some LA remained associated with HNF4α2, suggesting a relatively tight interaction (Figure 1C, top panel). Nonetheless, addition of exogenous LA to the lipid-depeleted media resulted in a ∼3-fold increase in the amount of LA bound (peak intensity 2.2×10^7^ v. 8.6×10^6^ without LA) (Figure 1C, middle). Although we could force this increase in LA binding, we did not observe an increase in binding of a related fatty acid (palmitoleic acid, PLA, C16:1) provided under similar conditions (Figure 1C, bottom). These findings further demonstrate that HNF4α does not effectively associate with other fatty acids when expressed in mammalian cells.

To further confirm binding specificity, we sought to create an orthologous receptor-ligand pair that does not bind endogenous LA. As noted above, the guanidinium group of R226 makes direct contact with the carboxylic head group of fatty acids (Figure 1D, left) [31]. We asked whether an artificial receptor-ligand pair could be established by reversing this charge interaction – i.e., by converting R226 to a negatively charged glutamate residue (R226E) whose carboxylate group is predicted by molecular modeling to interact with the amine moiety of linoleamide (LAA), a positively charged amide derivative of LA (Figure 1D, right). Indeed, AIMS analysis verified that R226E bound LAA in cells, whereas binding to LA could not be detected (Figure 1E). Taken together, these findings demonstrate the lipid binding specificity of HNF4α and suggest that LA binds HNF4α via the R226 guanidinium-carboxylate interaction.

### HNF4a ligand binding is reversible in mammalian cells

**Figure 2:**
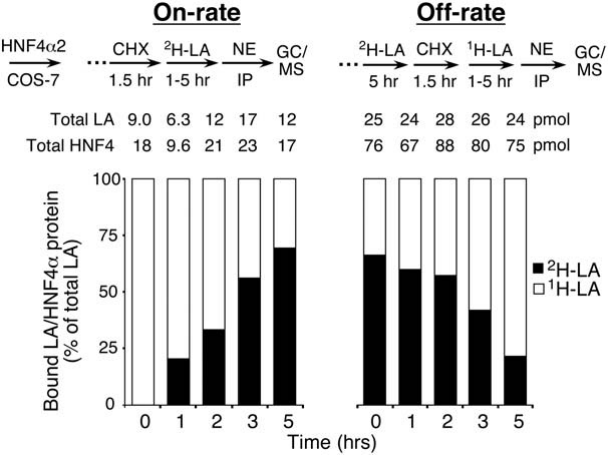
Binding of LA to HNF4α2 is exchangeable in mammalian cells. The on- and off-rate of LA binding to rat HNF4α2 in mammalian cells was determined by incubating COS-7 cells transfected with HNF4α2 wt with [^2^H]LA (30 µM) for 1 to 5 hr after (on-rate) or before (off-rate) a 1.5-hr treatment with cycloheximide (50 µM, CHX). [^1^H]LA (open) and [^2^H]LA (filled) bound to HNF4α2 was determined as in Figure 1 and graphed as a percent of the total bound LA normalized to the amount of HNF4α2 protein in the immunoprecipitate. Absolute amounts of LA and HNF4α2 protein are noted above each bar.

Previous studies suggested that a variety of fatty acids bind in a non exchangeable fashion to bacterially expressed HNF4α and HNF4γ [30], [31]. The inability to exchange with free fatty acids would exclude the type of “on/off” regulatory switch that is characteristic of ligand-modulated nuclear receptors. If so, the fatty acid might serve more like a co-factor than a hormone [41]. However, these conclusions were based on *in vitro* studies using HNF4 expressed in bacterial environments where protein folding, redox state and lipid milieu differ dramatically from the native mammalian environment. Thus, we utilized the AIMS technique to determine the ability of bound LA to freely exchange within mammalian cells. On-rate experiments were performed by adding [^2^H]LA to cycloheximide-treated cells (Figure 2, left). In the absence of new HNF4α2 synthesis, we found that within 5 hours more than half the endogenous [^1^H]LA that bound to existing HNF4α2 was replaced with exogenous [^2^H]LA. Conversely, off-rate experiments showed that pre-bound [^2^H]LA was replaced with exogenously added [^1^H]LA within a similar time frame (Figure 2, right). These results indicate that LA is an exchangeable ligand that binds native HNF4α in a reversible fashion.

### The HNF4α ligand binding pocket is unoccupied in the livers of fasted animals

**Figure 3:**
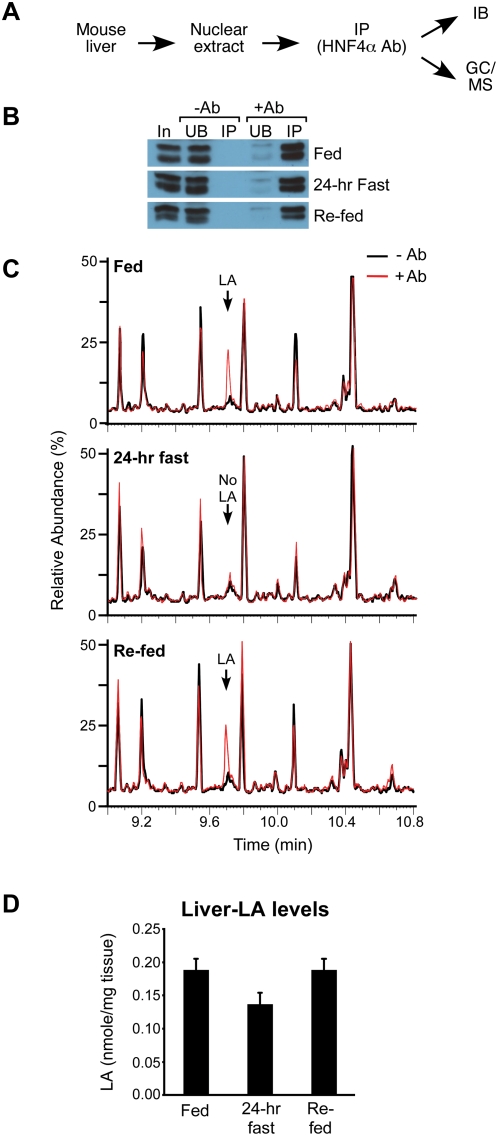
Native hepatic HNF4α binds endogenous LA in fed but not fasted mice. (A) Design: As in Figure 1A except with livers from male C57BL/6 mice fed a standard diet (Fed), fasted for 24 hr (24-hr Fast) or fasted for 24 hr and re-fed for 24 hr (Re-fed). (B) HNF4α from hepatic nuclei was IP'd in the absence (−Ab) or presence (+Ab) of the HNF4α-specific antibody and quantified by IB. HNF4α resolves as a doublet in this gel system. In, 2% of total input; UB, 2% of unbound material; IP, 2% of IP'd material. (C) GC/MS chromatograms (9 to 11 min) comparing compounds extracted from IP'd material from fed, fasted and re-fed animals with (red) or without (black) HNF4α Ab. Arrow, LA peak. (D) Quantification of the amount of LA in the liver of fed, 24-hr fasted and re-fed mice using GC/MS. Shown are average amounts of LA (nmole/mg liver) from 6 mice +/− SEM per group. Statistics: two-tailed t-test: fed vs. fasted, p = 0.057; fasted vs. re-fed, p = 0.051; fed vs. re-fed, ns; ANOVA p = 0.0641.

We next sought to determine what ligands may bind HNF4α *in vivo* under relevant physiological conditions. Livers were removed from young adult male C57BL/6 mice that were either fed a standard diet (fed), fasted for 24 hrs (24-hr fast), or fasted for 24 hrs followed by refeeding (re-fed). Hepatic HNF4α was then IP'd from isolated nuclei and quantified by immunoblot (IB) analysis (Figure 3A and 3B). GC/MS analysis of the isolated protein demonstrated that HNF4α bound LA in both the fed and re-fed state but not after a 24-hr fast (Figure 3C). No other fatty acid was found bound to HNF4α even when dietary LA was depleted by fasting, further confirming a preference of HNF4α for LA. Gel shift analysis showed that HNF4α protein from livers of fasted animals binds DNA well, indicating that the LA-free HNF4α retains DNA-binding function (Supplementary Figure S2). The decrease in HNF4α-bound LA was surprising since the total free fatty acid pool increases in fasting livers [42]. However, LA did not increase in 24-hr fasted livers; if anything, LA levels decreased by ∼27% in fasted vs. fed or re-fed livers (Figure 3D). This is consistent with the dietary requirement for LA and may at least partially explain the decrease in HNF4α occupancy in fasted mice (Figure 3C). These findings demonstrate that LA is bound to native HNF4α *in vivo*, and that binding is a physiological marker of the fed, but not the fasted, state.

### Function of HNF4α ligand binding

**Figure 4:**
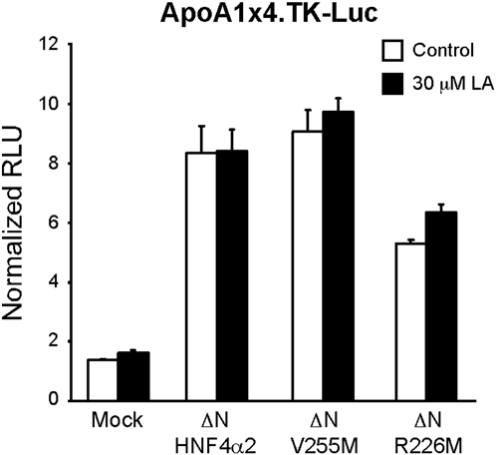
HNF4α exhibits ligand-independent transcriptional activity. Transient transfection into CV-1 cells maintained in stripped serum with rat HNF4α2 wt and LBP mutants V255M and R226M lacking the N-terminal AF-1 domain (ΔN-HNF4α2, ΔN-V255M, ΔN-R226M), reporter ApoA1x4.TK-Luc and co-activator PGC1α in the absence (Control) and presence of exogenously added LA (30 µM). Shown are relative light units (RLU) normalized to β-gal activity +/− SEM. Statistics: Bonferroni's multiple comparison post-test: control vs LA, ns for all constructs; p<0.001 for mock vs. all constructs; ANOVA p<0.0001.

For classical receptor-ligand pairs, ligand binding induces a conformational change that results in co-regulator recruitment and subsequent transcriptional modulation. Therefore, many previous attempts at ligand identification for orphan receptors utilized transcriptional activation and/or coactivator recruitment as the primary screen. However, unlike AIMS, these approaches are biased in that they can only identify ligands that induce transcriptional activity. Nonetheless, once we had identified LA in AIMS as binding HNF4α, it seemed reasonable to determine whether LA could transcriptionally activate HNF4α2 in transfected CV-1 cells exposed to lipid-depleted medium. No positive effect of LA was observed on full length HNF4α2 with or without expression of PGC1α (data not shown), a key coactivator that mediates HNF4α activity in the liver [43]. Since lack of apparent LA-responsiveness could reflect the high constitutive activity mediated by the ligand-independent AF-1 domain [44], we next examined N-terminally deleted (ΔN) HNF4α2 constructs that lack the AF-1. LA also had no effect on ΔN-HNF4α2 in the presence (Figure 4A) or absence (data not shown) of PGC1α. Similarly, significant LA-responsiveness was not observed on other well-characterized HNF4α responsive promoters (ApoB, PEPCK, G6Pase, data not shown). These findings provide further support for the utility of the AIMS approach since LA cannot be identified as an HNF4α ligand by commonly used transcriptional screening strategies.

The apparent lack of LA responsiveness could reflect a constitutive activity that is inherent to ligand-free HNF4α. To explore this possibility, we examined the transcriptional activity of the ΔN derivatives of the two non-LA binding HNF4α mutants (ΔN-V255M and ΔN-R226M) identified in Figure 1B. Since both mutants retained significant constitutive activity in the presence of PGC1α (Figure 4: V255M, 100% activity; R226M, 63%), we conclude that unoccupied HNF4α can activate transcription. Similar experiments with HNF4α R226E and its orthologous ligand LAA were not interpretable as R226E does not bind DNA well (data not shown). Nonetheless, the HNF4α mutants that were tested demonstrate that LA-free HNF4α, which is characteristic of the fasted state, retains significant transcriptional activity (Figure 4 and data not shown).

Despite the clear ligand-independent transactivation observed in the transfection assays on select target genes, it remained possible that ligand binding could modulate HNF4α activity on other target genes that we did not examine. It has been well established for other NR-ligand pairs that ligands can have different effects on different promoters [9], [45]–[47]. Furthermore, we hypothesized that LA might have a more pronounced effect on endogenous targets with the appropriate chromatin structure. Therefore, we used a genome-wide approach to ask whether there are any endogenous HNF4α target genes that are affected by exogenously added LA. We first verified by GC/MS that we could sufficiently deplete tissue culture cells and HNF4α of endogenous LA by incubating the cells in serum free media for 60 hr (Supplementary Figure S3). Then we used a recombinant adenovirus system to ectopically express HNF4α in a human colon cancer cell line, HCT116, which does not express endogenous HNF4α protein (Supplementary Figure S4); since HNF4α is normally expressed in the colon, this cell line should provide the appropriate environment to examine the effect of HNF4α on endogenous target genes. The adenovirus system also allowed for a prolonged incubation in serum free media in order to maximally deplete the cells of LA, while still being able to express HNF4α (Supplementary Figure S4).

**Figure 5:**
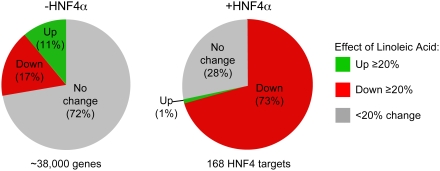
Genome-wide expression profiling of HNF4α in the presence and absence of LA. HCT116 cells infected with a tetracycline-inducible recombinant adenovirus expressing rat HNF4α1 (Adeno.ratHNF4α1.VSV) were incubated for 60 hr in serum free media in the absence (DMSO) or presence of 30 µM LA. The experiment, consisting of four experimental conditions (−HNF4 −LA, −HNF4 +LA, +HNF4 −LA, +HNF4 +LA), was performed in biological triplicate. All samples had doxycycline and the Tet-On virus; samples without HNF4α (−) and with HNF4α (+) differed only in infection with Adeno.ratHNF4α1.VSV. Pie charts, results of Affymetrix expression profiling. Effect of LA (≥20% change) on all genes in the absence of HNF4α (left); effect of LA (≥20% change) on 168 genes up regulated ≥3-fold by HNF4α (right).

**Figure 6:**
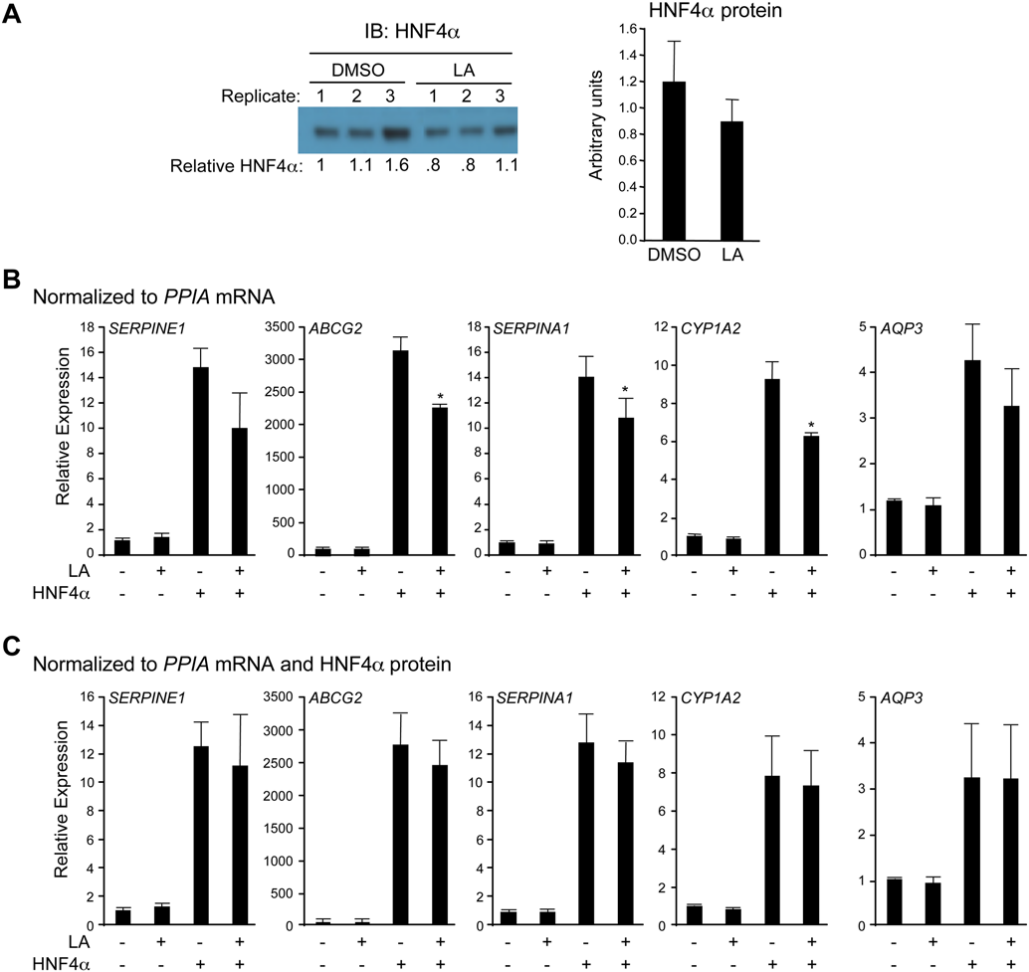
Effect of LA on HNF4α protein levels and target gene expression. Experimental design as described in Figure 5. (A) One of five representative IBs performed on whole cell extracts of adenovirus-infected HCT116 cells (left) and average quantification of all five blots normalized to Coomassie stain (right). (B,C) qRT-PCR results normalized to cyclophilin A (*PPIA*) mRNA (B) or to *PPIA* mRNA and HNF4α protein levels (C) of select HNF4α targets. The average qRT-PCR result from technical triplicates of each biological sample was normalized to the appropriate HNF4α protein level. Error bars represent standard deviation of the biological plus technical replicates. Statistics: two-tailed t-test, +HNF4−LA vs. +HNF4+LA. *, p<0.05.

We performed expression profiling using Affymetrix whole human genome Gene Chips on infected cells incubated in the absence or presence of 30 µM LA for 60 hrs. The results show that whereas the majority of the genes (72%) were not affected by the addition of LA in the absence of HNF4α (Figure 5, left pie chart), among the 168 genes that were constitutively activated 3-fold or more by HNF4α expression, ∼73% were down regulated 20% or more by the addition of LA. Interestingly, only two HNF4α target genes were up regulated 20% or more by LA (Figure 5) (see Supplementary Table S1 for a list of HNF4α targets affected by LA). We next examined the effect of LA on several individual HNF4α target genes by quantitative real time PCR (qRT-PCR) and again observed a modest decrease in target gene expression, verifying the array results (Figure 6B). However, we also noticed that there was a modest but consistent decrease in the amount of HNF4α protein in the samples containing LA (Figure 6A). Therefore, we normalized each individual qRT-PCR result to its appropriate HNF4α protein level and found that the decrease by LA was no longer significant (Figure 6C). These results confirm that ligand-free HNF4α has considerable constitutive transactivation activity and that the presence of LA does not enhance that activity. If anything, the presence of LA seemed to modestly repress the constitutive activity of HNF4α, although that effect may be mediated by an alteration in the HNF4α protein level.

## Discussion

Orphan nuclear receptors are central regulators of a wide range of physiological and pathological events. Over the past ∼20 years, a number of orphan receptors have been “de-orphanized,” yet some of those ligand assignments may not represent the actual ligand that is bound to the receptor *in vivo* – i.e., the true endogenous ligand (TEL). Identification of synthetic or non-endogenous ligands remains critical as those compounds serve as important experimental tools and provide critical mechanistic insights into the function of nuclear receptors. Nonetheless, a complete understanding of receptor-regulated networks requires knowledge of the TELs as well as how the TELs are generated and what their affect may be on receptor activity. Existing experimental strategies are not specifically designed to identify TELs and are often biased by an implicit assumption that the TEL is contained within a particular compound library or biological extract. Our work establishes the AIMS approach as a viable strategy to identify TELs based purely on their association with a receptor *in vivo*: no pre-assumptions are required as to the nature of the ligand. AIMS is likely to be applicable to virtually all NRs as the only requirement is an antibody, or other means to selectively isolate the receptor. Perhaps the only limitation to this approach is the ability to obtain sufficient amounts of native receptor to ensure that the cognate ligand is within the detection limits of the assay.

In addition to identifying endogenous ligands, the AIMS approach has the unique advantage of being a function-independent assay. Many of the existing screening technologies are biased by the assumption that the ligand functions as a transcriptional modulator – this excludes the possibility of identifying NR ligands that selectively modulate non-genomic NR functions such as kinase activation, proteasomal degradation and intracellular trafficking, etc. [48]–[51]. Indeed, the existence of some TELs may have been overlooked because they would not have been detected by transcription- or coactivator-based screens. HNF4α is a case in point: here we use the AIMS approach to demonstrate that LA is the endogenous ligand bound to rat, mouse and human HNF4α (Figures 1,2,3 and S3) and that addition of LA fails to alter the activation by the receptor (Figures 4,5,6).

### HNF4α is transcriptionally active in the absence of ligand

We also show here that during fasting native HNF4α exists in an LA-free state (Figure 3), and that the ligand-free HNF4α-PGC1α complex is transcriptionally active (Figure 4). The ligand-independent transcriptional activity that is characteristic of HNF4α in the fasted state is consistent with the physiology of the fasted liver. Indeed, hepatic gluconeogenesis is stimulated by fasting-induced expression and recruitment of PGC1α to gluconeogenic HNF4α target genes [43]. Ligand-independent activity is also consistent with structural studies that show that the position of the HNF4α AF-2 is altered primarily by binding of co-factor peptides [52]. Thus, our finding of transcriptional activity for ligand-free HNF4α is consistent with the known structural and physiological activities of this receptor.

### HNF4-LA, a primitive receptor-ligand pair?

The origin of NRs and their ligands is a hotly debated topic (see [34] and references therein), and one that can benefit from experimental approaches such as the AIMS assay. There are basically two camps, one that proposes that the ancestral NR was unliganded (e.g., [53]) and the other that proposes that NRs always had ligands (often referred to as the ligand exploitation hypothesis) (e.g., [54]). Since HNF4 is one of the few NRs found in primitive metazoans such as sponge and coral reef [37], [38], it could be viewed as an ancient receptor and possibly even one of the founders of the superfamily. As such, it is tempting to speculate on the significance of our findings on the evolution of receptor-ligand pairs. There is evolutionary logic to the notion that a relatively simple lipid (such as LA) would have been available to ancient organisms and could thus serve as a primordial ligand [55]. Binding of more complex ligands (e.g., steroids, retinoids, etc.) may have been a later development associated with more recently evolved receptor proteins. If that is the case, and if the more ancient forms of HNF4 also bound LA, then the results presented here could impact the discussion of the origins of ligand-dependent transcription. One possibility is that the modern HNF4α•LA pair accurately reflects the primitive receptor-ligand pair, which would suggest that ligand binding activity was acquired in advance of the ability to modulate transcription. It would also suggest that subsequent receptor-ligand pairs acquired the AF-2-mediated conformational switch associated with transcriptional activation later on during evolution. If so, HNF4 could represent a “missing-link” in the evolutionary pathway from a non ligand-regulated transcription factor to a transcription factor that can both bind and respond to ligand. The alternative hypothesis is that ligand binding and ligand-regulated transcription appeared at the same time for HNF4 but ligand-regulated transcription was subsequently lost at some point during the evolution to modern mammalian HNF4α. Additional studies are clearly required to appropriately address these important questions; the application of the AIMS assay to ancient NRs should prove useful in this regard.

### Is there a non transcriptional function for the HNF4α ligand?

It remains possible that the HNF4 ligand used to have, and/or continues to have, an as yet-to-be determined function that is distinct from transcriptional modulation. The physiological linkage of receptor occupancy to the fasting/fed state raises the possibility that the ligand contributes to a non-genomic mechanism that affects this physiological transition. Potential non-genomic activities that have been linked to other nuclear receptors include kinase activation, proteasomal degradation and intracellular trafficking, etc. [48]–[51]. The identification of LA as an endogenous HNF4α ligand will open up new avenues of research that explore the potential for LA to modulate non-transcriptional activities. This demonstrates the power and utility of the AIMS approach that identifies ligands based on binding rather than preconceived notions of function.

### Resurrection of HNF4α as a drug target?

Previous studies suggested that a wide variety of fatty acids are associated with bacterially expressed HNF4 [30], [31], though LA was not among these. In retrospect, this is undoubtedly due to the fact that *E. coli* do not have LA [56]. Although HNF4α•LA interactions were not examined in those studies, the authors mention that the fatty acids they did observe bound to HNF4 could not be removed without denaturing the protein. This in turn led to the notion that synthetic HNF4α ligands would have little utility as they would not be able to compete with a non exchangeable endogenous ligand. Hence, HNF4α, despite its many links to human disease, was no longer deemed a suitable drug target. In sharp contrast, we show here that when HNF4α is expressed in its native environment, it specifically and reversibly associates with one predominant fatty acid: LA. We also show that under certain physiological conditions, the LBP of HNF4α is unoccupied indicating that ligand binding is specifically linked to physiological status. These findings suggest that HNF4α may indeed be a tractable target for small molecule drug discovery. The predicted function of such synthetic ligands, however, will benefit from an understanding of the functional role of the endogenous ligand.

## Materials and Methods

### Ethics Statement

Care and treatment of experimental animals was in accordance with guidelines from the University of California, Riverside, Institutional Animal Care and Use Committee (IACUC).

### Affinity Isolation/Mass-Spectrometry (AIMS)

Nuclear extracts containing 2–5 µg (40–100 pmoles) of HNF4α protein (two to three mouse livers or two 150-mm plates of COS-7 cells transfected with rat HNF4α2) were incubated with 30–45 µg of HNF4α antibody (Ab) (α445, [20]) for 2 hr at 4°C. (See Supporting Information Materials and Methods S1 for detailed methods on preparation of nuclear extracts. Experimental conditions required the use of rat, mouse and human HNF4α for different experiments. These receptors are very highly conserved at the sequence and functional levels as discussed in Supplementary Figure S5.) Protein A Sepharose beads (50 µl of a 1∶1 suspension in PBS) (Pierce) were added and incubated at 4°C for 4 hr with gentle rocking. The beads were washed three times at room temperature with 500 µl sterile, filtered PBS by inverting 7–10 times and pelleting at 1000× *g* for 10 min. During the last wash, 2% or 4% of the beads were sampled and HNF4α was detected by IB analysis with either HNF4α Ab conjugated to HRP (Peroxidase Labeling Kit, Roche Pharmaceuticals) or HNF4α Ab followed by TrueBlot HRP-conjugated antibody (eBiosciences). Both methods avoid detection of the IgG used in the IP. The gas chromatography and mass spectrometry (GC/MS) protocol was adapted from Yuan and Forman [57]. Specifically, IP'd material (protein plus beads) in PBS was heated at 65°C for 30 min. The beads were pelleted by low speed centrifugation and the supernatants were filtered and extracted with two to three volumes of ethyl acetate three times. Organic fractions were dried under N_2_ gas and derivatized with N,O,-bis (trimethylsilyl) trifluoacetamide containing 1% trimethylchorosilane (Pierce) at 55°C for 6 hr, and subsequently analyzed with a ThermoFinnigan Trace DSQ GC/MS system run in scanning mode over a *m/z* range of 50–700. Following data acquisition, total ion spectra from the samples were compared to known spectra contained in the NIST 98 Library using the Finnigan Xcalibur software. Gas chromatographic separation was performed with Phenomenex ZB-5 (5% phenyl-95% dimethyl-polysiloxane) column (15 or 30 meters, 0.25 mm ID, film thickness 0.50 µm). At the start of each run, the column temperature was kept at 50°C for 1 min, increased using a gradient of 25°C per min up to 300°C, and held at 300°C for an additional 8 min (15-meter column) or 15 min (30-meter column). Difference in retention time of LA is due to different column lengths; standards were run on all columns.

### *In vivo* Exchange Assay

Rat HNF4α2 cDNA (NM_022180) (pMT7.rHNF4α2) was introduced into COS-7 cells as described in Supporting Information. Cells were maintained in DMEM supplemented with 10% bovine calf serum and penicillin/streptomycin throughout the experiment. Nuclear extraction, IP, IB and GC/MS analysis of all samples were performed as described above and in Supporting Information. *On-rate experiment* - To halt *de novo* protein synthesis, cells were treated with 50 µM cycloheximide (Sigma) 32 hr after transfection. After 1.5 hr, fresh media containing 30 µM [^2^H]LA and 50 µM cycloheximide was added. After 0, 1, 2, 3, and 5 hr [^2^H]LA treatment, nuclear extracts were prepared, HNF4α2 was IP'd, and the associated ligand was analyzed by GC/MS and the amount of HNF4α2 protein recovered was determined by IB analysis as described above. *Off-rate experiment* - Approximately 37 hr after transfection, fresh media containing 30 µM [^2^H]LA was added and the cells were incubated at 37°C for 5 hr to allow for exchange with pre-bound, non-labeled endogenous LA. Cells were then treated with 50 µM cycloheximide for 1.5 hr. To remove the [^2^H]LA, the media was replaced with fresh media containing 30 µM non-labeled LA and 50 µM cycloheximide. Nuclear extracts were prepared after 0, 1, 2, 3 and 5 hr and analyzed as described above for the on-rate experiments.

### Mouse liver extraction for LA measurement

To determine the amount of LA in whole liver tissue of fed, fasted, and 24 hr fasted animals, mouse liver tissue (n = 6 per condition, ∼20 mg per liver) was homogenized in PBS, then extracted with 1.1 ml of ethyl acetate. PBS containing various concentrations of an LA standard were extracted simultaneously and used to establish a standard curve. Heptadecanoic acid (20 nmole, Sigma) was added to all samples as an internal control for extraction efficiency.

### Identification of candidate LA-responsive HNF4α target genes

Human colorectal carcinoma HCT116 cells (#CCL-247, ATCC) maintained in McCoy's 5A (Hyclone) media supplemented with 10% Tet System Approved fetal bovine serum (Clontech) and penicillin/streptomycin at 37°C and 5% CO_2_ were grown to 50–60% confluency at which point the media was changed to McCoy's 5A, penicillin/streptomycin plus 0.15% fatty acid-free BSA (EMD Biosciences) plus vehicle (0.1% DMSO) or 30 µM linoleic acid (LA) (Sigma). The cells were then immediately co-infected with 2.5 MOI each of the Adeno-X Tet-On virus (Clontech) and the recombinant adenovirus expressing VSV-tagged rat HNF4α1 under Tet control [58]. (HNF4α1 differs from HNF4α2 by 10 amino acids in the F domain; both activate transcription well although HNF4α2 is somewhat more responsive to co-activators than HNF4α1 [59]. The DNA binding domain of rat and human (and mouse) HNF4α is 100% identical and hence these species are anticipated to have very similar if not identical DNA binding specificity (see Supplementary Figure S5). At 12 hr post infection, 0.5 µg/ml doxycycline (Sigma) was added to induce expression of HNF4α and the cells were maintained under serum-free conditions with vehicle or 30 µM LA for an additional 48 hr (60 hr total in serum-free conditions with vehicle or LA). All cells received the same amount of doxycycline and Tet-On virus, but only those cells also infected with the Adeno.ratHNF4α1.VSV expressed HNF4α protein. RNA isolated from one-half of three biological replicate plates using the RNeasy Mini kit (Qiagen) was pooled and hybridized to the GeneChip Human Genome U133 plus 2.0 (Affymetrix) as per the manufacturer's protocol in the Genomics Core in the UCR Institute for Integrated Genome Biology. Each pooled RNA sample was analyzed on two separate arrays and the results were averaged (8 arrays total for the four different conditions – +/−LA +/−HNF4). Whole cell extracts harvested from the other half of the plate [60] were used to verify HNF4α protein levels by IB analysis.

### Analysis of Array Data

All arrays were analyzed using GC Robust Multi-array Average (GCRMA) background adjustment and quantile normalization on probe-level data sets with R software (http://www.bioconductor.org). When comparing two categories (i.e., +/−LA or +/−HNF4) we included only those probe sets for which at least one treatment group had 100% Present (P) or Marginal (M) calls (determined by the MAS5 algorithm), as has been previously described [61].

### Quantitative Real-Time PCR

Quantitative real-time PCR (qRT-PCR) analysis was used to verify the relative expression of select genes, with preference given to either known HNF4α targets [21], or genes that contained promoters previously indicated in the literature to bind HNF4α in human cells [22], [62]. Genomic DNA was removed by DNAse I treatment at 37°C for 1 hr and the RNA was reverse transcribed using random hexamers and SuperScript III (Invitrogen). The resulting cDNA was diluted and analyzed using a MyiQ single-color real-time PCR detection system (Bio-Rad) and SYBR Green. Gene-specific primers (see Supplementary Table S2) were validated over four orders of magnitude and analyzed with the iQ5 Optical System Software (Bio-Rad); primer pairs were deemed valid if an input log plot amount versus C_T_ generated an efficiency of 100%±10% and a correlation coefficient of *R^2^* = 0.950±0.05. Each biological triplicate was analyzed in technical triplicate. The relative expression level of the genes was evaluated using the Pfaffl method [63], normalizing to cyclophilin A (*PPIA*) expression.

## Supporting Information

Supporting Materials and Methods S1 Reagents, plasmids, ectopic expression of HNF4α proteins in COS-7 cells, preparation of mouse liver nuclear extracts, immunoblot (IB) analysis, reporter gene assay, molecular modeling are described in detail.
(0.09 MB DOC)

Figure S1 Binding of LA to HNF4α2 occurs within cells. Deuterated LA was added to nuclear extracts before IP and subsequent GC/MS. The ratio of [^1^H]LA to [^2^H]LA shows that the LA that is bound to HNF4α2 is derived from endogenous [^1^H]LA.
(0.07 MB PDF)

Figure S2 HNF4α from fasted mouse liver binds DNA. EMSA of nuclear extracts from the livers of fed and 24-hr fasted mice showing that HNF4α from fasted animals binds DNA, indicating that the apoHNF4α is functional.
(1.25 MB PDF)

Figure S3 Validation of experimental system for LA candidate gene identification I. GC/MS of endogenous HNF4α IP'd from human tissue culture cells (Hep3B) incubated in media without serum +/− LA for 60 hr showing that LA can be depleted from the cells and HNF4α.
(0.23 MB PDF)

Figure S4 Validation of experimental system for LA candidate gene identification II. IB analysis of extracts from human colon cancer cells HCT116 infected with recombinant adenovirus expressing HNF4α showing a lack of expression of endogenous HNF4α and a robust expression of the recombinant HNF4α.
(6.90 MB PDF)

Figure S5 Alignment of human, mouse and rat HNF4α2 amino acid sequence. Alignment of the amino acid sequence of these receptors shows the high degree of similarity, especially in the DBD and LBD, including the residues that contact that the ligand.
(0.12 MB PDF)

Table S1 Effect of linoleic acid on HNF4α target genes (Affymetrix array results).
(0.08 MB PDF)

Table S2 Primers used for qRT-PCR to verify HNF4α target genes.
(0.06 MB PDF)
