# Supplementary material for: Identification of an Endogenous Ligand Bound to a Native Orphan Nuclear Receptor
Source: PLoS One. 2009 May 19;4(5):e5609. doi: 10.1371/journal.pone.0005609 (PMC2680617; doi:10.1371/journal.pone.0005609)
Supplement: Supporting Materials and Methods S1 — Reagents, plasmids, ectopic expression of HNF4α proteins in COS-7 cells, preparation of mouse liver nuclear extracts, immunoblot (IB) analysis, reporter gene assay, molecular modeling are described in detail. (0.09 MB DOC) [file pone.0005609.s001.doc]

**SUPPORTING INFORMATION**

**Identification of an Endogenous Ligand Bound to a Native Orphan Nuclear Receptor**

Xiaohui Yuan^*^, Tuong Chi Ta^*^, Min Lin, Jane R. Evans, Yinchen Dong, Eugene Bolotin, Mark A. Sherman, Barry M. Forman^#,§^, Frances M. Sladek^#,§^

^*,#^Contributed equally

^§^To whom correspondence should be addressed: [frances.sladek@ucr.edu](mailto:frances.sladek@ucr.edu) or [bmforman@earthlink.net](mailto:bmforman@earthlink.net)

**This file contains:**

Supporting Material and Methods and References

**See separate files for:**

Supplementary Figure S1

Supplementary Figure S2

Supplementary Figure S3

Supplementary Figure S4

Supplementary Figure S5

Supplementary Table S1

Supplementary Table S2

**SUPPORTING MATERIALS AND METHODS**

**Reagents**

The affinity purified antibody to HNF4α (HNF4α Ab) used in the immunoprecipitations (IPs) and immunoblots (IB) has been previously described (α445 in [1]). Linoleic acid (LA) (C18:2; *cis, cis*-9,12 octadecadienoic acid) (Sigma), palmitoleic acid [C16:1; (*Z*)-9-hexadecenoic acid] (Sigma), linoleamide (LAA) (Biomol), and deuterated linoleic acid (^2^H-LA) (Cayman Chemical) were prepared as 30 mM stocks in ethanol or DMSO. All fatty acids were protected from light, stored at -20 or -80°C under nitrogen or argon gas and diluted to a final concentration of 30 μM. The appropriate vehicle was used at 0.1%.

**Plasmids**

The expression vector pMT7.rHNF4α2 containing wild type (wt) rat HNF4α2 (NM_022180), the predominant isoform in liver [2], was generated by inserting the rat HNF4α2 cDNA, generously provided by S. Hata (Kyoto University, Japan), into the Bam HI site in pMT7 [3]. Ligand binding pocket (LBP) point mutations valine (Val, V) to methionine (Met, M) at residue 255 (V255M), arginine (Arg, R) to Met at residue 226 (R226M), and Arg to glutamic acid (Glu, E) at residue 226 (R226E) were generated in pMT7.rHNF4α2 with the QuikChange XL kit (Stratagene). N-terminally truncated mutants were generated from these pMT7 vectors by PCR amplification of the cDNA starting at aa 43 (9 residues N-terminal to the first cysteine (Cys) of the zinc finger), adding a Met residue and subcloning into pCMX-PL1 vectors with Bam HI and Xho I [4]. The PGC1α expression vector containing mouse PGC1α (aa 1-794) with a C-terminal Myc epitope and 6 × His tag in pcDNA3.1-myc-his(-)B (Invitrogen) was kindly provided by D. Kelly (Washington University, St. Louis). The HNF4α reporter construct (ApoA1x4.Tk-Luc) contains four HNF4α binding sites from the human apolipoprotein A1 (*APOA1*) promoter (‘site A’, 5’-GGGGTCAAGGGTT-3’ at -194 to -206) upstream of the HSV.TK promoter driving expression of the firefly Luciferase (Luc) gene. Human PGC1α (NP_032930) (aa 91 to 408) fused to GST (pGEX.HA-PGC1α) was kindly provided by A. Kralli (Scripps Research Institute, La Jolla) [5].

**Ectopic expression of HNF4α proteins in COS-7 cells**

COS-7 cells (ATCC CRL-1651), maintained at 37°C and 5% CO2 in Dulbecco’s modified Eagle’s medium (DMEM) (CellGro) supplemented with 10% bovine calf serum (Hyclone) lipid-depleted serum (“Stripped Serum”) [Controlled Process Serum Replacement (CPSR3), Sigma] and penicillin/streptomycin (CellGro), were transiently transfected with pMT7.rHNF4α2 via calcium phosphate precipitation as previously described [6]. Where applicable, 30 μM of exogenous fatty acids were added 12-24 hr after transfection. Cells were harvested after additional 24-34 hr incubation. Nuclear extracts were prepared as previously described [6]. “Mock” transfected samples contained either the pMT7 empty vector or no DNA.

**Preparation of mouse liver nuclear extracts**

Mouse liver nuclear extracts were prepared using a combination of previously published methods [1,7]. Briefly, C57BL/6 male mice (9-10 weeks old, Harlan Bioproducts for Science), maintained on a strict 12-hr light/dark cycle and fed a standard lab chow (LabDiet #5001) *ad libitum*, were euthanized by CO2 asphyxiation. The liver was removed, rinsed in PBS*,* minced and then homogenized in Buffer A [10 mM HEPES (pH 7.8), 0.32 M sucrose, 0.3% Triton X-100, 25 mM KCl, 0.15 mM spermine, 0.5 mM spermidine, 1 mM EGTA, 1 mM EDTA, 1 mM DTT, 0.5 mM PMSF] containing protease and phosphatase inhibitor*s* (P8340, P2850, P5726, Sigma) with an electric tissue grinder. The homogenate was passed through a cell strainer, homogenized manually and centrifuged at 2000 × *g* for 10 min. The nuclear pellet was washed in Buffer A followed by two washes in Low Salt Buffer (Buffer A except without sucrose or Triton-X but with 20% glycerol) and resuspended in Low Salt Buffer. High Salt Buffer (Low Salt Buffer except with 0.5 M KCl), was added for a final salt concentration of 0.33 M KCl and the protein was extracted by gentle agitation for 45-60 min*.* The debris was pelleted 14,000 × *g*, 20 min and the supernatant (nuclear extract) was removed, aliquoted, snap frozen on dry ice and stored at -80°C. All steps were carried out at 4°C. Protein concentration was determined by the BioRad Protein Assay (BioRad). For fasted animals, food was removed 24 hr prior to euthanasia. Re-fed animals were fasted for 24 hr before re-feeding the standard lab chow for 24 hr. Care and treatment of experimental animals was in accordance with guidelines from the University of California, Riverside, Institutional Animal Care and Use Committee (IACUC).

**Immunoblot (IB) Analysis**

The amount of HNF4α protein in COS-7 and mouse liver nuclear extracts was determined by semi-quantitative immunoblot (IB) analysis. Crude nuclear extracts (5-20 μg total protein) were analyzed by 10% SDS-PAGE, transferred to Immobilon-P (Millipore) and probed with HNF4α Ab as previously described [8], followed by detection with ECL Western Detection Reagent (GE HealthCare/Amersham). The signal from the nuclear extracts was quantified (NIH Image J software) relative to a recombinant HNF4α standard containing the targeted epitope (aa 444 to 455 of HNF4α1): GST.LBD/F expressed in bacterial cells and purified using GSH agarose as previously described [9].

**Reporter Gene Assay**

CV-1 cells (ATCC# CCL-70) maintained in charcoal-resin stripped fetal bovine serum (Gemini) were transfected in 96-well plates as previously described [10]. HNF4α expression vectors (0.7 to 0.3 ng/well), pcDNA3.1mPGC1α co-activator (3 ng/well), reporter construct ApoA1x4-TK.Luc (35 ng/well) and CMV β-galactosidase (β-gal) control (58 ng/well) were transfected into cells by lipofection using DOTAP (Roche). Cells were exposed to ligands or vehicle (DMSO, 0.1%) for 40 hr and then harvested and assayed for luciferase and β-gal activity. All transfections were performed in multiples of six and repeated four or more times. Shown are results from one representative experiment with SD of the six samples normalized to β-gal activity.

**Molecular modeling**

A molecular model of LA bound to the LBD of HNF4α was built using the crystal structure of rat HNF4α bound to lauric acid (C12:0, dodecanoic acid) as a template [PDB file 1M7W, [11]]. The positions of the additional six carbons in LA were optimized using a combination of molecular dynamics and energy minimization (Discover 3.0/CVFF forcefield, Accelrys, San Diego) until they completely filled the upper arm of the internal L-shaped binding cavity which is lined by residues Cys179 (helix 3), Ile349 (helix 11), Ala355 (loop), Ile357 (loop), and Leu361 (helix 12), the latter being the first residue of the LXXLL AF-2 consensus motif. The atoms of the protein and the first 12 carbon atoms of the ligand were immobilized during the simulation. A molecular model of linoleamide (LAA) docked in the active site of the R226E mutant form of rat HNF4α was built by modifying and minimizing the wt model until the newly introduced amide group formed a hydrogen bond with the side chain carboxylate of Glu226 (H to O distance = 2.11 Å).

**Supporting References**

1. Sladek FM, Zhong WM, Lai E, Darnell JE, Jr. (1990) Liver-enriched transcription factor HNF-4 is a novel member of the steroid hormone receptor superfamily. Genes Dev 4: 2353-2365.

2. Hata S, Tsukamoto T, Osumi T (1992) A novel isoform of rat hepatocyte nuclear factor 4 (HNF-4). Biochim Biophys Acta 1131: 211-213.

3. Jiang G, Sladek FM (1997) The DNA binding domain of hepatocyte nuclear factor 4 mediates cooperative, specific binding to DNA and heterodimerization with the retinoid X receptor alpha. J Biol Chem 272: 1218-1225.

4. Umesono K, Murakami KK, Thompson CC, Evans RM (1991) Direct repeats as selective response elements for the thyroid hormone, retinoic acid, and vitamin D3 receptors. Cell 65: 1255-1266.

5. Teyssier C, Ma H, Emter R, Kralli A, Stallcup MR (2005) Activation of nuclear receptor coactivator PGC-1alpha by arginine methylation. Genes Dev 19: 1466-1473.

6. Jiang G, Nepomuceno L, Hopkins K, Sladek FM (1995) Exclusive homodimerization of the orphan receptor hepatocyte nuclear factor 4 defines a new subclass of nuclear receptors. Mol Cell Biol 15: 5131-5143.

7. Costa RH, Lai E, Grayson DR, Darnell JE, Jr. (1988) The cell-specific enhancer of the mouse transthyretin (prealbumin) gene binds a common factor at one site and a liver-specific factor(s) at two other sites. Mol Cell Biol 8: 81-90.

8. Maeda Y, Seidel SD, Wei G, Liu X, Sladek FM (2002) Repression of hepatocyte nuclear factor 4alpha tumor suppressor p53: involvement of the ligand-binding domain and histone deacetylase activity. Mol Endocrinol 16: 402-410.

9. Ruse MD, Jr., Privalsky ML, Sladek FM (2002) Competitive cofactor recruitment by orphan receptor hepatocyte nuclear factor 4alpha1: modulation by the F domain. Mol Cell Biol 22: 1626-1638.

10. Forman BM, Tontonoz P, Chen J, Brun RP, Spiegelman BM, et al. (1995) 15-Deoxy-delta 12, 14-prostaglandin J2 is a ligand for the adipocyte determination factor PPAR gamma. Cell 83: 803-812.

11. Dhe-Paganon S, Duda K, Iwamoto M, Chi YI, Shoelson SE (2002) Crystal structure of the HNF4 alpha ligand binding domain in complex with endogenous fatty acid ligand. J Biol Chem 277: 37973-37976.
