# Supplementary material for: Identification of an Endogenous Ligand Bound to a Native Orphan Nuclear Receptor
Source: PLoS One. 2009 May 19;4(5):e5609. doi: 10.1371/journal.pone.0005609 (PMC2680617; doi:10.1371/journal.pone.0005609)
Supplement: Figure S1 — Binding of LA to HNF4α2 occurs within cells. Deuterated LA was added to nuclear extracts before IP and subsequent GC/MS. The ratio of [1H]LA to [2H]LA shows that the LA that is bound to HNF4α2 is derived from endogenous [1H]LA. (0.07 MB PDF) [file pone.0005609.s002.pdf]

LA bound to HNF4α2 (pmole)	
[^{1}H]LA	[^{2}H]LA
7.8	ND

Figure S1. Binding of LA to HNF4α2 occurs within cells.
COS-7 cells were transfected with rat HNF4α2 wt and cultured in medium with stripped serum. LA binding to HNF4α2 was determined as in Fig. 1 in the main text except that nuclear extracts were prepared with lysis buffer containing 30 μM [^{2}H]LA. [^{1}H]LA-specific ions (*m/z* 262 and 337) and [^{2}H]LA-specific ions (*m/z* 266 and 341) of the silylated derivative of LA were used for quantification by GC/MS. The detection limit for [^{2}H]LA is less than 1 pmole. These results demonstrate that the LA that is bound to HNF4α2 is derived from endogenous [^{1}H]LA within the cell, not from the exogenous [^{2}H]LA that is unique to the lysis and extraction procedures. ND, not detectable.
