# Supplementary material for: Identification of an Endogenous Ligand Bound to a Native Orphan Nuclear Receptor
Source: PLoS One. 2009 May 19;4(5):e5609. doi: 10.1371/journal.pone.0005609 (PMC2680617; doi:10.1371/journal.pone.0005609)
Supplement: Figure S2 — HNF4α from fasted mouse liver binds DNA. EMSA of nuclear extracts from the livers of fed and 24-hr fasted mice showing that HNF4α from fasted animals binds DNA, indicating that the apoHNF4α is functional. (1.25 MB PDF) [file pone.0005609.s003.pdf]

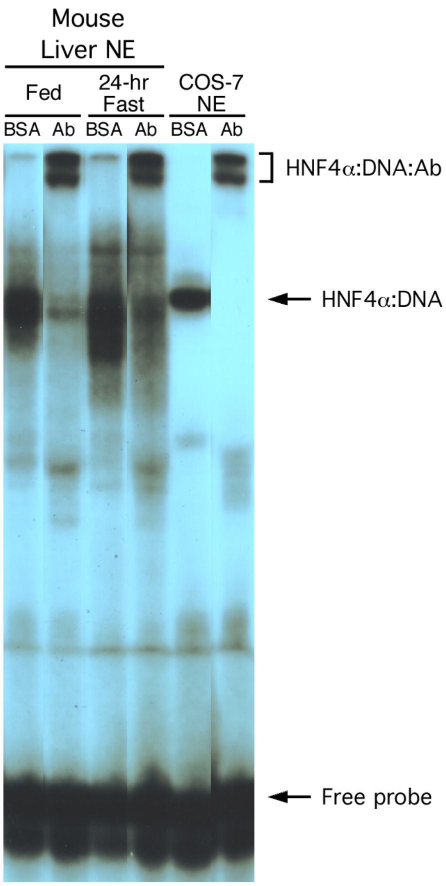

Figure S2. HNF4α from fasted mouse liver binds DNA.
EMSA (electrophoretic mobility shift assay) using nuclear extract (NE) prepared from fed (Fed) and fasted (24-hr Fast) mouse liver (10 μg total protein containing approximately 10 ng HNF4α as determined by immunoblot analysis with a recombinant standard, not shown) and wild-type rat HNF4α2-transfected COS-7 cells on a ^{32}P-labeled probe from the human ApoA1 promoter (5'-AGGGCAGGGGTCAAGGGTTCAGT-3', -210 to -188). See Materials and Methods in main text for details on NE preparation and EMSA conditions [1]. Total protein was normalized with bovine serum albumin (BSA). The α445 antibody (Ab, 1 μg) was added as indicated. The HNF4α2:DNA and HNF4α:DNA:Ab complexes and free probe are indicated. The lanes shown were reassembled from a single gel/exposure.

1. Jiang G, Nepomuceno L, Hopkins K, Sladek FM (1995) Exclusive homodimerization of the orphan nuclear receptor hepatocyte nuclear factor 4 defines a new subclass of nuclear receptors. Mol Cell Bio 15: 5131-5143.
