# Supplementary material for: Identification of an Endogenous Ligand Bound to a Native Orphan Nuclear Receptor
Source: PLoS One. 2009 May 19;4(5):e5609. doi: 10.1371/journal.pone.0005609 (PMC2680617; doi:10.1371/journal.pone.0005609)
Supplement: Figure S3 — Validation of experimental system for LA candidate gene identification I. GC/MS of endogenous HNF4α IP'd from human tissue culture cells (Hep3B) incubated in media without serum +/− LA for 60 hr showing that LA can be depleted from the cells and HNF4α. (0.23 MB PDF) [file pone.0005609.s004.pdf]

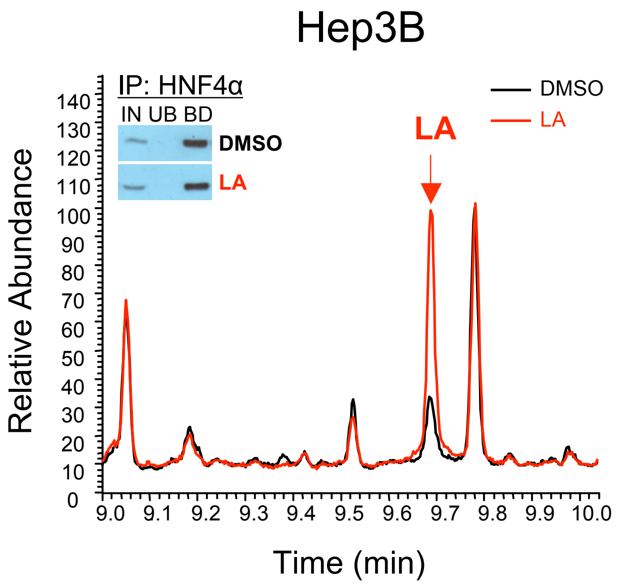

Figure S3. Validation of experimental system for LA candidate gene identification I.

GC/MS chromatogram and immunoblot (inset) showing HNF4α immunoprecipitated (IP) from human hepatocellular carcinoma cells (Hep3B) cells after a 60-hr incubation in serum free media (DMEM plus fatty acid-free BSA) in the absence (DMSO) or presence of 30 μM linoleic acid (LA). Inset: IN, input; UB, unbound; BD, bound. See main text for details on methods. The results show that these tissue culture conditions can effectively decrease the amount of LA bound to HNF4α protein.
