# Supplementary material for: Identification of an Endogenous Ligand Bound to a Native Orphan Nuclear Receptor
Source: PLoS One. 2009 May 19;4(5):e5609. doi: 10.1371/journal.pone.0005609 (PMC2680617; doi:10.1371/journal.pone.0005609)
Supplement: Figure S4 — Validation of experimental system for LA candidate gene identification II. IB analysis of extracts from human colon cancer cells HCT116 infected with recombinant adenovirus expressing HNF4α showing a lack of expression of endogenous HNF4α and a robust expression of the recombinant HNF4α. (6.90 MB PDF) [file pone.0005609.s005.pdf]

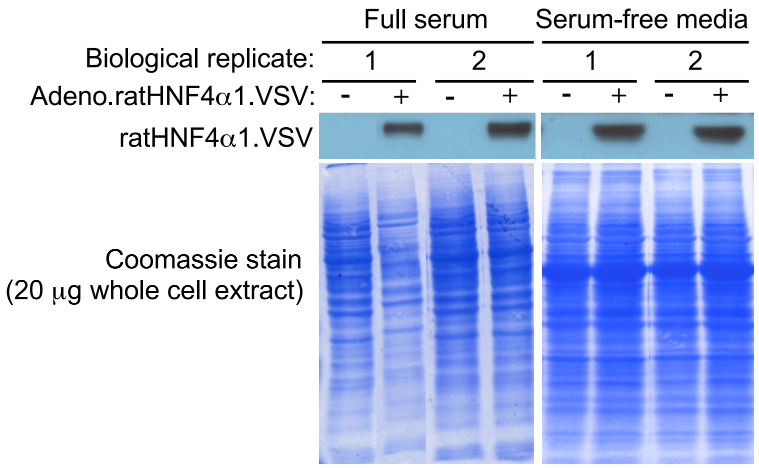

Figure S4. Validation of experimental system for LA candidate gene identification II. Immunoblot analysis of whole cell extracts showing a lack of endogenous HNF4α in HCT116 cells and a robust expression of HNF4α in cells infected with recombinant Adeno.ratHNF4α1.VSV incubated under the indicated media conditions. Coomassie stain (bottom panel) shows equal loading. Lanes are reassembled from two gels as indicated.
