# Supplementary material for: Identification of an Endogenous Ligand Bound to a Native Orphan Nuclear Receptor
Source: PLoS One. 2009 May 19;4(5):e5609. doi: 10.1371/journal.pone.0005609 (PMC2680617; doi:10.1371/journal.pone.0005609)
Supplement: Figure S5 — Alignment of human, mouse and rat HNF4α2 amino acid sequence. Alignment of the amino acid sequence of these receptors shows the high degree of similarity, especially in the DBD and LBD, including the residues that contact that the ligand. (0.12 MB PDF) [file pone.0005609.s006.pdf]

Human, mouse and rat HNF4α2 amino acid sequence alignment

```
                 1                                                      56
Human   MRLSKTLVDMDMADYSAALDPAYTTLEFENVQVLTMGNDTSPSEGTNLNAPNSLGVSALCAICGD
        •••••••  •••••••••••••••••••••••••••••••••••• •••  ••••••••••••••
Mouse   MRLSKTLAGMDMADYSAALDPAYTTLEFENVQVLTMGNDTSPSEGANLNSSNSLGVSALCAICGD
        •••••••• ••••••••••••••••••••••••••••••••••••••••••••••••••••••••
Rat     MRLSKTLADMDMADYSAALDPAYTTLEFENVQVLTMGNDTSPSEGANLNSSNSLGVSALCAICGD

                                                                        121
Human   RATGKHYGASSCDGCKGFFRRSVRKNHMYSCRFSRQCVVDKDKRNQCRYCRLKKCFRAGMKKEAV
        •••••••••••••••••••••••••••••••••••••••••••••••••••••••••••••••••
Mouse   RATGKHYGASSCDGCKGFFRRSVRKNHMYSCRFSRQCVVDKDKRNQCRYCRLKKCFRAGMKKEAV
        •••••••••••••••••••••••••••••••••••••••••••••••••••••••••••••••••
Rat     RATGKHYGASSCDGCKGFFRRSVRKNHMYSCRFSRQCVVDKDKRNQCRYCRLKKCFRAGMKKEAV

                                 H1                                H3   186
Human   QNERDRISTRRSSYEDSSLPSINALLQAEVLSRQITSPVSGINGDIRAKKIASIADVCESMKEQL
        •••••••••••••••••••••••••••••••• ••••• ••••••••••••• • ••••••••••
Mouse   QNERDRISTRRSSYEDSSLPSINALLQAEVLSQQITSPISGINGDIRAKKIANITDVCESMKEQL
        •••••••••••••••••••••••••••••••••••••••••••••••••••••••••••••••••
Rat     QNERDRISTRRSSYEDSSLPSINALLQAEVLSQQITSPISGINGDIRAKKIANITDVCESMKEQL

                                H4-H5      R226                         251
Human   LVLVEWAKYIPAFCELPLDDQVALLRAHAGEHLLLGATKRSMVFKDVLLLGNDYIVPRHCPELAE
        •••••••••••••••• ••••••••••••••••••••••••••••••••••••••••••••••••
Mouse   LVLVEWAKYIPAFCELLLDDQVALLRAHAGEHLLLGATKRSMVFKDVLLLGNDYIVPRHCPELAE
        •••••••••••••••••••••••••••••••••••••••••••••••••••••••••••••••••
Rat     LVLVEWAKYIPAFCELLLDDQVALLRAHAGEHLLLGATKRSMVFKDVLLLGNDYIVPRHCPELAE

          V255      H7            H8                         H9         316
Human   MSRVSIRILDELVLPFQELQIDDNEYAYLKAIIFFDPDAKGLSDPGKIKRLRSQVQVSLEDYIND
        ••••••••••••••••••••••••••• •••••••••••••••••••••••••••••••••••••
Mouse   MSRVSIRILDELVLPFQELQIDDNEYACLKAIIFFDPDAKGLSDPGKIKRLRSQVQVSLEDYIND
        •••••••••••••••••••••••••••••••••••••••••••••••••••••••••••••••••
Rat     MSRVSIRILDELVLPFQELQIDDNEYACLKAIIFFDPDAKGLSDPGKIKRLRSQVQVSLEDYIND

                    H10-H11                       H12                   381
Human   RQYDSRGRFGELLLLLPTLQSITWQMIEQIQFIKLFGMAKIDNLLQEMLLGGSPSDAPHAHHPLH
        •••••••••••••••••••••••••••••••••••••••••••••••••••••• ••••• ••••
Mouse   RQYDSRGRFGELLLLLPTLQSITWQMIEQIQFIKLFGMAKIDNLLQEMLLGGSASDAPHTHHPLH
        •••••••••••••••••••••••••••••••••••••••••••••••••••••••••••• ••••
Rat     RQYDSRGRFGELLLLLPTLQSITWQMIEQIQFIKLFGMAKIDNLLQEMLLGGSASDAPHAHHPLH

                                  α2 insert                             446
Human   PHLMQEHMGTNVIVANTMPTHLSNGQMCEWPRPRGQAATPETPQPSPPGGSGSEPYKLLPGAVAT
        ••••••••••••••••••• •••••••••••••••••••••••••••• ••••• •••••••  •
Mouse   PHLMQEHMGTNVIVANTMPSHLSNGQMCEWPRPRGQAATPETPQPSPPSGSGSESYKLLPGAITT
        •••••••••••••••••••••••••••••••••••••••••••••••••••••••••••••••••
Rat     PHLMQEHMGTNVIVANTMPSHLSNGQMCEWPRPRGQAATPETPQPSPPSGSGSESYKLLPGAITT

                          465
Human   IVKPLSAIPQPTITKQEVI
        •••• •••••••••••• •
Mouse   IVKPPSAIPQPTITKQEAI
        •••••••••••••••••••
Rat     IVKPPSAIPQPTITKQEAI
```

Figure S5. Alignment of human, mouse and rat HNF4α2 amino acid sequence.
In this study, we determined the ligand bound to three different species of HNF4α – human (Fig. S3), mouse (Fig. 3) and rat (Fig.s 1,2). The ligand binding domain (LBD) of rat and mouse HNF4α is 100% identical on the amino acid level. The LBD of rat/mouse HNF4α is 97.5% identical to human; there are only 6 amino acids that are different, none of which contact the ligand [1,2]. The DNA binding domain (DBD) of human, mouse and rat HNF4α are 100% identical. Indicated is the conventional amino acid numbering for HNF4α2, which does not include an N-terminal extension resulting from an alternative translation start site (underline) [3]. HNF4α1 (used in Fig.s 5,6) is identical to HNF4α2 except for the α2 insert (double underline). Highlight: black, species differences; red, ligand contacts; yellow, DBD; green, LBD helices; blue, LBD β-sheets. Residues mutated in this study (V255, R226) are noted. Human HNF4α2 (NM_000457.3); mouse HNF4α2 (NM_008261); rat HNF4α2 (NM_022180).

1. Dhe-Paganon S, Duda K, Iwamoto M, Chi YI, Shoelson SE (2002) Crystal structure of the HNF4 alpha ligand binding domain in complex with endogenous fatty acid ligand. J Biol Chem 277: 37973-37976.
2. Wisely GB, Miller AB, Davis RG, Thornquest AD, Jr., Johnson R, et al. (2002) Hepatocyte nuclear factor 4 is a transcription factor that constitutively binds fatty acids. Structure 10: 1225-1234.
3. Sladek FM, Zhong WM, Lai E, Darnell JE, Jr. (1990) Liver-enriched transcription factor HNF-4 is a novel member of the steroid hormone receptor superfamily. Genes Dev 4: 2353-2365.
