# Supplementary material for: Identification of an Endogenous Ligand Bound to a Native Orphan Nuclear Receptor
Source: PLoS One. 2009 May 19;4(5):e5609. doi: 10.1371/journal.pone.0005609 (PMC2680617; doi:10.1371/journal.pone.0005609)
Supplement: Table S1 — Effect of linoleic acid on HNF4α target genes (Affymetrix array results). (0.08 MB PDF) [file pone.0005609.s007.pdf]

Table S1. Effect of linoleic acid on HNF4α target genes (Affymetrix array results)

Gene Symbol	Fold Δ by HNF4	% Δ by LA
**Up by LA**		
MYB	3.7	+32.7
VIPR1	3.6	+27.0
**Down by LA**		
APOBEC1	102.2	-23.3
NR0B2	66.9	-53.2
HBA2	39.5	-43.8
HBA1	32.8	-38.4
ABCG2	**24.4**	**-41.2**
LOC646927	21.5	-39.8
SERPINE1	**21.2**	**-49.4**
CYP1A2	**17.2**	**-47.6**
SEC14L2	15.3	-25.6
SERPINA1	**14.3**	**-29.7**
SPHK1	12.1	-65.5
RTP3	11.7	-38.7
DHRS3	10.9	-24.7
SEPP1	10.8	-23.4
C6orf117	10.4	-23.2
ACSL5	10.0	-23.4
PKD2L1	9.2	-64.9
CALML4	9.2	-20.0
GDA	8.2	-35.8
CYP4F12	8.2	-64.7
AQP3	**7.6**	**-30.3**
BIN2	7.6	-42.9
TCF7	7.5	-47.3
PDK4	7.2	-29.6
DIRAS3	7.2	-30.6
KLK6	6.4	-22.9
ARPM1	6.3	-63.5
PLCXD1	6.2	-23.8
ANKRD41	5.9	-34.2
SIRPA	5.7	-26.1
KIAA1509	5.3	-24.1
IL32	5.2	-69.8
MGC7036	5.1	-23.2
KCNQ4	5.1	-29.7
C6orf141	4.9	-33.6
OGG1	4.5	-30.2
TRIM10	4.4	-51.0
CDKN2C	4.3	-27.2
KIAA1727	4.3	-21.5
MLXIPL	4.2	-29.9
**Down by LA (cont.)**		
CA2	4.1	-27.2
ABP1	4.1	-44.6
KLK7	4.1	-27.9
SRG	4.1	-28.5
TRIM6	4.1	-20.8
DSP	4.0	-27.5
DGAT2	4.0	-41.7
CTNS	4.8	-33.5
CRLF3	4.8	-41.3
ROR1	4.8	-30.1
RBM20	4.6	-26.5
TMEM116	4.0	-35.6
NAGS	4.0	-28.1
CTSZ	4.0	-28.1
DNM2	3.9	-36.0
TRIM15	3.9	-24.6
KRT7	3.9	-29.3
FAM110C	3.7	-26.9
ZNF557	3.6	-40.1
C20orf112	3.6	-30.3
PQLC2	3.6	-59.2
PIPOX	3.6	-25.6
TJP3	3.6	-34.3
NKX3-1	3.5	-39.9
DKFZp666G057	3.5	-67.8
FOXO1A	3.5	-29.5
KCNK5	3.5	-53.5
SLC16A5	3.4	-47.4
RBKS	3.4	-37.4
HTRA2	3.4	-27.8
VPS13D	3.4	-28.5
FAM101B	3.3	-27.7
SDC1	3.3	-21.6
LOC646762	3.3	-47.3
CYP24A1	3.3	-25.7
NSUN6	3.2	-26.9
PNPLA2	3.2	-23.9
STAP2	3.2	-54.5
ZNF207	3.1	-21.4
LOC116236	3.1	-40.0
TUBAL3	3.1	-36.5
C1orf93	3.1	-27.2
RBPMS2	3.0	-27.9
FBXL15	3.0	-39.1

Genes up-regulated >3.0-fold in human colorectal cancer (HCT116) cells by Adeno.ratHNF4α1.VSV with >20% change by LA and raw intensity values >50 (+HNF4 -LA); results are from expression profiling using human Affymetrix U133 2.0 arrays (average of duplicate arrays from biological triplicates). See main text for detailed methods. Genes in bold were verified by qRT-PCR (see Fig. 6 in main text).
