# Supplementary material for: Identification of an Endogenous Ligand Bound to a Native Orphan Nuclear Receptor
Source: PLoS One. 2009 May 19;4(5):e5609. doi: 10.1371/journal.pone.0005609 (PMC2680617; doi:10.1371/journal.pone.0005609)
Supplement: Table S2 — Primers used for qRT-PCR to verify HNF4α target genes. (0.06 MB PDF) [file pone.0005609.s008.pdf]

Table S2. Primers used for Quantitative Real-Time PCR to verify HNF4α target genes

Gene	GenBank Accession Number	Projected Amplicon Length	Forward Primer 5'->3'	Reverse Primer 5'->3'	Notes[1]
SERPINA1	NM_001002236	134 bp	GTCAAGGACACCGAGGAAGA	TATTTCATCAGCAGCACCCA	QPPD
SERPINE1	NM_000602	150 bp	CCATGCTCCAGCTGACAAC	TGGTGCTGATCTCATCCTTG	Across exons 2-3
CYP1A2	NM_000761	63 bp	GGGCACTTCGACCCTTACAA	GCACATGGCACCAATGACG	QPPD
AQP3	NM_004925	211 bp	GACAGAAGGAGCTGGTGTCC	ATGAGGATGCCCAGAGTGAC	Across exons 1-2
ABCG2	NM_004827	229 bp	CCTGAGATCCTGAGCCTTTG	ATGCCTTCAGGTCATTGGAA	Across exons 1-2
PPIA	NM_021130	116 bp	CCCACCGTGTTCTTCGACAT	CCAGTGCTCAGAGCACGAAA	QPPD

[1]Primer pairs were either selected from among the published sets within the Quantitative PCR Primer Database (QPPD, http://web.ncifcrf.gov/rtp/gel/primerdb/default.asp) or designed to span exon-exon boundaries using Primer3 (http://frodo.wi.mit.edu/).
